# Determination of Internal Temperature by Measuring the Temperature of the Body Surface Due to Environmental Physical Factors—First Study of Fever Screening in the COVID Pandemic

**DOI:** 10.3390/ijerph192416511

**Published:** 2022-12-08

**Authors:** Izabela Gorczewska, Agnieszka Szurko, Agnieszka Kiełboń, Agata Stanek, Armand Cholewka

**Affiliations:** 1 Department of Nuclear Medicine and Endocrine Oncology, Maria Sklodowska-Curie National Research Institute of Oncology, 44-102 Gliwice, Poland; 2 Faculty of Science and Technology, University of Silesia, 75 Pułku Piechoty 1A, 41-500 Chorzów, Poland; 3 Department and Clinic of Internal Medicine, Angiology and Physical Medicine, Faculty of Medical Sciences in Zabrze, Medical University of Silesia, Batorego 15, 41-902 Bytom, Poland

**Keywords:** thermovision, noncontact fever detection, COVID-19, the core temperature of the body

## Abstract

The SARS-CoV-2 virus pandemic has shown that the use of a contact thermometer to verify the elevated body temperature of a suspected person carries a risk of spreading disease. The perfect solution seems to be the use of thermal imaging as a diagnostic method in fever evaluation. The aim of the research is to develop an algorithm for thermovision measurements in fever screening standards in the context of the impact of various weather conditions on the temperature of people entering the public institution. Each examined person had two thermal images of the face—AP and lateral projection. Using a T1020 FLIR thermal camera with a resolution of 1024 × 768 pixels; the mean temperature was measured from the area of the forehead, the maximum forehead, the corners of the eyes, the inside of the mouth and the external auditory canal temperature. On the other hand, using classic contact thermometers, the temperature in the armpit and ear was measured. The obtained preliminary results showed very strong and positive correlations between the temperature in the ear measured with an ear thermometer and the maximum, minimum and average forehead temperature. These correlations oscillate at approximately r = 0.6, but the highest value of Spearman coefficient was obtained for the mean temperature of the forehead. Moreover, high correlations were also obtained between the temperature in the ear, measured with an ear thermometer, and the maximum temperature in the corners of the eyes and in the ear, measured with a thermal imaging camera. These values were, respectively, r = 0.54, r = 0.65. In summarizing, remote body temperature measurement taken with a thermal camera can be useful in the assessment of the body’s core temperature.

## 1. Introduction

Coronavirus disease (COVID-19) is an infectious disease caused by the SARS-CoV-2 virus. After the first case of coronavirus disease in Wuhan, China in late December 2019, Acute Respiratory Syndrome 2 (SARS-CoV-2) coronavirus spread to over 200 countries in approximately 3 months [1]. The WHO states in its reports that after a consistent decline since the end of January 2022, the number of new cases per week increased by 8% in the week of 7–13 March 2022. As of 13 March 2022, there have been over 455 million confirmed cases and over 6 million reported deaths [2].

There are four types of COVID-19 patients: asymptomatic, mild, moderate and severe. Research shows that over 80% of COVID-19 cases have mild symptoms, but 10–20% of COVID-19 cases go into a severe stage [3]. Elderly people, as well as those with cardiovascular disease, diabetes, chronic respiratory disease, or cancer, are at greater risk of developing severe disease [1,4,5,6], while in cancer patients, recent prospective studies have shown that severe forms of COVID-19 are not directly related to cancer and cancer treatment but can be attributed to multiple comorbidities and poor overall health in this patient population [7,8].

Fever, dry cough, diarrhea, difficulty breathing (dyspnoea), headache, and pneumonia are the most common symptoms of COVID-19 [9]. 

Body temperature monitoring is essential, especially for the early detection of COVID-19 suspects. A fever is said to be when the body temperature is 38 °C or higher. On the other hand, low-grade fever occurs when the temperature is between 37 °C and 38 °C [10]. When body temperature is higher than a certain level of thermoregulation, then physiological mechanisms lead to heat loss. This is manifested by an decrease in skin temperature as a result of dilation of blood vessels or/and increased sweating. These mechanisms work until a certain level of thermoregulation is achieved, i.e., the so-called state of balance [11].

The SARS-CoV-2 virus pandemic has shown that there are often situations where the use of a contact thermometer is difficult due to the measurement time required and the risk of disease transmission through contact. Of course, disinfection of the thermometer provides protection against possible infection with the COVID-19 virus. However, the use of classic thermometers works mainly in situations where a small group of people is tested. When it is necessary to carry out a quick screening test on a large group of people, the use of thermal imaging seems to be the ideal solution. The lack of contact with the examined person and the immediate measurement result are the main arguments in favor of using thermography to check whether a person’s temperature is elevated. Screening thermography systems have been deployed at airports since the SARS outbreak in Southeast Asia. They have become the basic method of screening febrile people in mass population centers. In addition, mass thermography of people with suspected fever at hospital entrances has become a standard [12,13,14,15,16]. However, it should be emphasized that despite the number of advantages of the thermography method, special attention should be paid to factors that hinder the interpretation of the results and the measurement itself, i.e., the climatic conditions in which the person is staying immediately before the measurement (e.g., before entering a hospital or public institution). The influence of humidity, air temperature or wind force on the temperature reading from the corner of the eyes, ears or forehead seems to be significant, leading to an increase/decrease in the temperature of exposed places. As a consequence, this may result in an incorrect assessment of the temperature of the tested person. So far, no one has taken into account the influence of atmospheric conditions on the temperature measurement in such systems.

The aim of the research is to develop an algorithm for performing thermographic measurements and to develop standards for screening for fever in various weather conditions in people/patients entering and leaving medical facilities and facilities.

Achieving the above goal will provide important information and help in the development of a fast, automatic screening method that allows for searching for people with an elevated body temperature in large groups of people. This will help to reduce the spread of a virus, such as SARS-COVID-19, as this non-contact method will significantly reduce examination time and enable early response and preventive measures.

## 2. Materials and Methods

These studies were carried out as part of the National Centre for Research and Development (NCBiR) project awarded as support for single-name hospitals. The University of Silesia in Katowice acted as a leader of the consortium. Therefore, the research was carried out in public institutions (buildings of the University of Silesia and Municipal Hospital No. 4 in Gliwice—a hospital adapted only to receive patients with SARS-CoV-2). All persons entering the buildings were invited to thermovision examinations and temperature measurements carried out with a classic thermometer. All 130 participants were screened with the same method. Therefore, no randomization was performed. Therefore, the research group consisted of adults of different sexes and ages. In addition, the presented results are preliminary and research is ongoing. Ultimately, the research group will consist of approximately 1000 people. The entire research project was approved by the Bioethics Committee of the Medical University of Silesia, number PCN/CBN/022/KB1/69/21, dated 15 May 2021.

The measuring equipment has been placed in a room near the entrance to the institution (inside building) and in such a way as to avoid the proximity of air conditioners or heaters. Measurements were carried out using the T1020 FLIR thermal imaging camera with a resolution of 1024 × 768 pixels.

Each examined person has two thermal images of the face—AP projection and lateral projection with a view of the external auditory canal taken. The camera lens was set at the level of the examined person’s face. Each time, the image was taken with the appropriate distance of the person from the lens, which was 50 cm +/− 5 cm. People with a fringe were asked to expose their foreheads while taking the measurement. Similarly, when taking pictures sideways, the subjects were asked to uncover their ear. In addition, each person wearing a mask, headband or glasses was asked to remove these items of clothing for the duration of the study. This information was also entered in the comments next to the measurement results.

**Figure 1:**
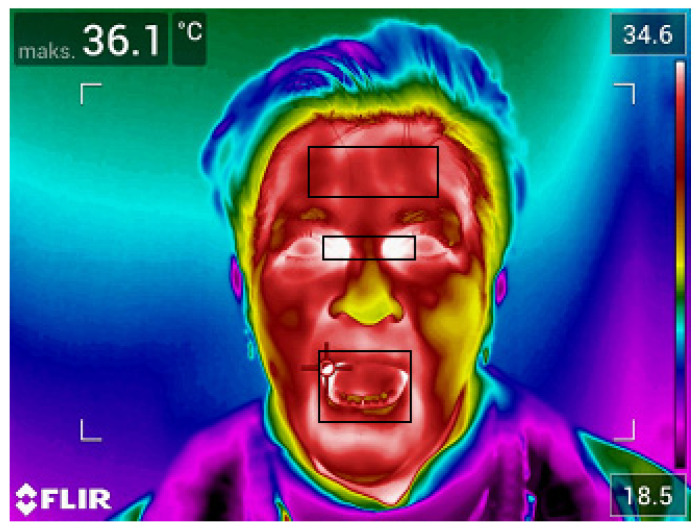
Thermal image of the face made in the anterior-posterior (AP) projection.

**Figure 2:**
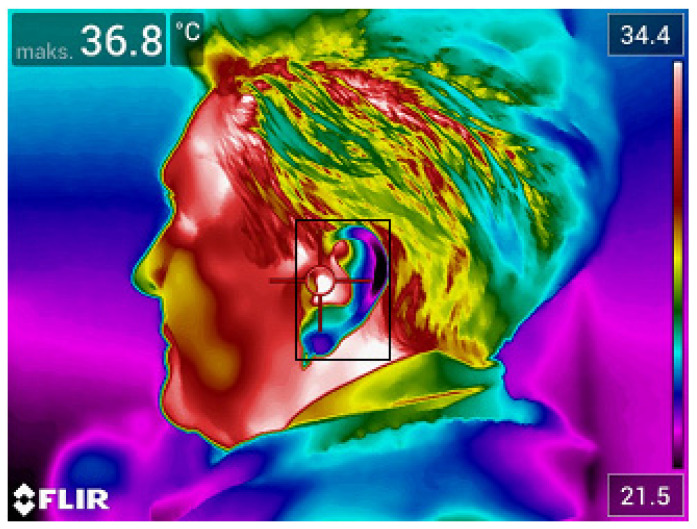
Thermal image of the face made in the lateral projection.

The temperature parameters (maximum and average temperature) were derived from regions of interest marked on the thermal images (forehead, inside the corners of the eyes, mouth and ear). Exemplary thermal images with marked regions of interest are presented in Figure 1 and Figure 2.

Measurements in the armpit and in the ear were measured with an electronic contact thermometer marked “clean”, i.e., thermometers that had been disinfected the previous day and placed in proper container. After each use, the thermometer was returned to the box labelled as used thermometers. At the end of the measurement day, they were disinfected and placed in a container with “clean” thermometers.

The analysis of the obtained results was carried out initially for 130 measurements. Statistical data analysis was performed in the Statistica StatSoft program. The collected data was correlated and evaluated using statistical tests to find a remote way to estimate core body temperature. The core temperature estimation will be a reliable method to identify people entering the institution who show fever and symptoms of the disease. For the purposes of the calculations, the level of statistical significance was assumed to be α = 0.05. Spearman correlation coefficients (r) were used to evaluate the relationships between temperature parameters and chosen external factors.

In the final stage of the measurements, it was necessary to determine the conditions characterizing the environment in which the thermographic test was carried out. Therefore, in addition to thermographic and thermometric measurements of people, it was necessary to measure the ambient temperature outside, wind speed and air humidity. These factors should be controlled during thermovision measurements and seems to contribute to showing the relationship between the body temperature measured remotely and the weather conditions of the test person before the measurement.

## 3. Results

**Figure 3:**
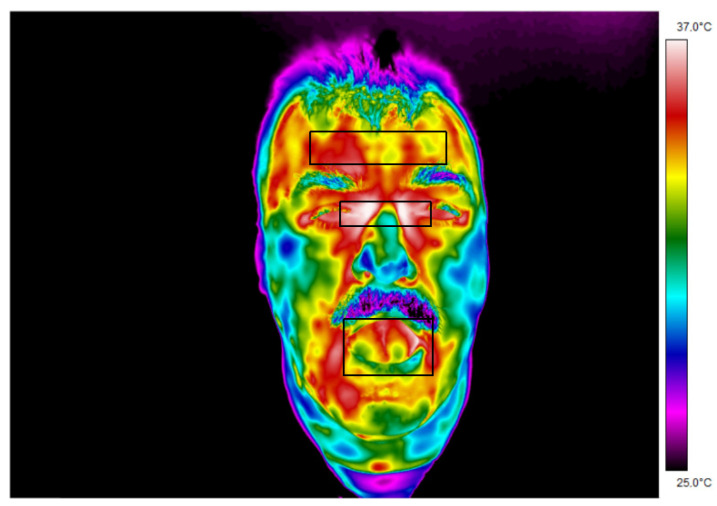
Thermogram 1 showing the temperature map of the subject’s facial surface.

**Figure 4:**
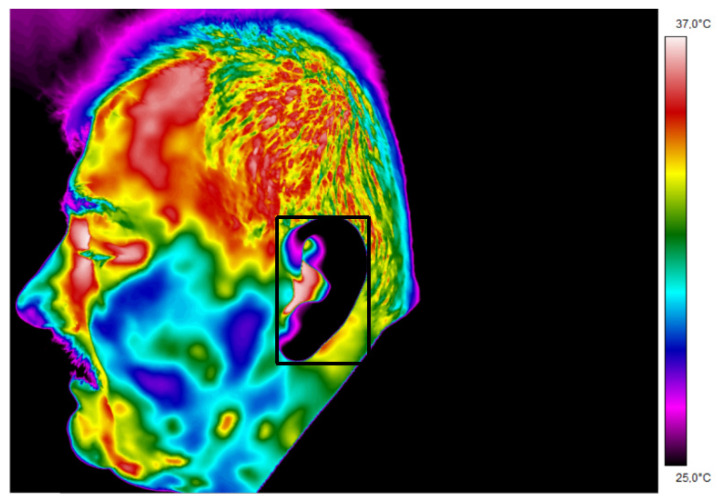
Thermogram 1 showing the temperature map of the subject’s lateral facial surface.

Examples of thermograms, with areas of interest, obtained for people entering the institution are presented below (Figure 3 and Figure 4).

The areas from which temperatures were read are marked on thermogram 1. It can be noticed that the highest surface temperature of the subject’s face is maintained near the corners of the eyes. In contrast, the cheeks and nose remain in the lower region of the temperature scale.

Thermogram 2 shows the temperature map of a face aligned with respect to the lens of the thermal camera in the AP projection. The area of interest containing the external auditory canal from which the maximum temperature was read was marked on the thermogram.

When analyzing the obtained thermograms, it can be noticed almost immediately that the temperature maps of people presented in the same temperature range have different temperature values, which is shown by different colors in the thermograms. These differences may be influenced not only by internal but also external factors.

Averaged values of temperature parameters obtained from the analysis of thermograms and the values of external factors are presented in the Table 1.

Additional correlations were also made between the temperatures in the ear and the armpit made with classic thermometers, with the temperatures of specific areas of the face read with a thermal imaging camera. The obtained results are presented in the tables below. Correlations that are statistically significant are marked in red. Correlation coefficients as well as *p*-values obtained in this experiment are listed in Table 2.

The highest significant positive correlation obtained between the measured temperatures and external factors occurs in the case of changes in outside air humidity and average temperature of the forehead of the examined person (r = 0.26, *p* = 0.003). It is worth mentioning that the forehead temperature values are statistically significantly dependent on all external factors (T_O_ vs. T_Fmin_ r = 0.25, *p* = 0.004; H_O_ vs. T_Fmin_ r = 0.25, *p* = 0.004; V_W_ vs. T_Fmin_ r = 0.25, *p* = 0.005; T_O_ vs. T_Fa_ r = 0.24, *p* = 0.006; H_O_ vs. T_Fa_ r = 0.26, *p* = 0.003; V_W_ vs. T_Fa_ r = 0.23, *p* = 0.0010). The other results were less consistent, as evidenced by little wicker correlation between external factors and temperatures under the armpit (T_O_ vs. T_AR_ r = 0, *p* = 0.999; H_O_ vs. T_AR_ r = −0.11, *p* = 0.215; V_W_ vs. T_AR_ r = −0.12, *p* = 0.192), in the corners of the eyes (T_O_ vs. T_CEmax_ r = 0.18, *p* = 0.039; H_O_ vs. T_CEmax_ r = 0.20, *p* = 0.215; V_W_ vs. T_CEmax_ r = 0.22, *p* = 0.013) and in the ear (T_O_ vs. T_Emax_ r = 0.22, *p* = 0.013; H_O_ vs. T_Emax_ r = 0.21, *p* = 0.017; V_W_ vs. T_Emax_ r = 0.16, *p* = 0.077), as well as between the measured temperature parameters (i.e., T_CEmax_ vs. T_AR_ r < 0.01, *p* = 0.991). Due to the weak level of correlations, external factors are considered to have little influence on the measurement of the core body temperature. According to the results presented in Table 3, the temperature in the armpit, measured with a classic thermometer, and the temperature in the ear, measured with an ear thermometer, did not depend on the external conditions. One might suppose that these parameters would be a good reflection of core body temperature. However, the temperature values from different measurement sites obtained with different methods were correlated and no significant correlations were found for all parameters with regard to the measurement of the armpit temperature. It was concluded that the temperature values measured in the ear with an ear thermometer will constitute the “gold standard” as all its correlations with other thermal parameters are statistically significant.

Therefore, the relationship between the human body temperature determined by a thermovision camera and the temperature value in the human ear measured with a standard ear thermometer was determined (Table 3).

It is assumed that the measurement of the temperature in the cavities of the human body are reliable places to determine the core temperature of the human body. Therefore, a strong correlation with the temperature measurements made in the oral cavity and ear was expected. Based on the obtained results, the highest correlation and high statistical significance is observed between the temperature in the ear measured with the ear thermometer and the maximum temperature in the ear determined from the analysis of thermograms (T_ET_ vs. T_Emax_ r = 0.65, *p* < 0.0001). On the other hand, changes in the temperature of the inside of the oral cavity in relation to the temperature in the ear (by the ear thermometer) were statistically significant, but the obtained Spearman correlation coefficient was low (T_ET_ vs. T_Mmax_ r = 0.27, *p* = 0.016).

High correlations exist also between the temperature measured in the ear with an ear thermometer and the average, maximum and minimum temperature of the forehead (Table 2). However, the obtained results clearly showed the highest correlation for the mean forehead temperature with the established gold standard (T_ET_ vs. T_Fa_ r = 0.62, *p* < 0.0001).

The maximum temperature measured at the corner of the eye also shows a significant and statistically strong correlation with the temperature measured in the ear with the ear thermometer (T_ET_ vs. T_CEmax_ r = 0.54, *p* < 0.0001). Thus, the corners of the eyes are the third area where the temperature measurement correlates with the designated gold standard.

The last analyzed place of temperature measurement was the armpit. Between the temperature measured in the armpit and the temperature inside the ear measured with an ear thermometer, this is the weakest, also statistically speaking, of the correlations obtained (T_ET_ vs. T_AR_ r = 0.15, *p* = 0.090).

In conclusion, the greatest correlations occurred between the temperature measured in the ear with an ear thermometer and the temperature measured in the ear with a thermal imaging camera (r = 0.65, *p* < 0.0001), as well as between the maximum temperature measured in the ear with an ear thermometer and the average temperature of the forehead (r = 0.62, *p* < 0.0001). These results show high positive correlations that are statistically significant.

## 4. Discussion

The aim of our research was to develop an algorithm for thermographic measurements and standards for screening for fever in various weather conditions.

Our thermal imaging studies have shown that the simplest and fastest measurement of human body temperature should take into account not only the maximum temperature of the corners of the eyes, but also the average temperature of the forehead or the maximum ear temperature. The performed statistical analysis clearly showed the link between these areas. The greatest correlations occurred for the temperature measured in the ear with a thermal imaging camera (r = 0.65, *p* < 0.0001) and the average temperature of the forehead (r = 0.62, *p* < 0.0001) in reference to the temperature in the ear (measured by an ear thermometer).

The temperature measured in the armpit with contact thermometers is a fairly common home method for assessing body temperature. Therefore, one might expect that the temperature measured in the armpit should give the same correlations as the temperature measured in the ear with an ear thermometer. Unexpectedly, in our study, the weakest correlation was between the temperature measured in the armpit and the temperature inside the ear measured with an ear thermometer (T_ET_ vs. T_AR_ r = 0.15, *p* = 0.090). The obtained result, contrary to the expected, makes it questionable whether the thermometers used give reliable results. It seems that the thermometers used had a large temperature drift, which could result from the short measurement time of approximately 5 s. Moreover, the error-prone temperature measurement under the armpit may be influenced by a number of factors, ranging from technical aspects to the measurement methodology, e.g., an inaccurately pressed thermometer to the armpit or high sweating.

High correlations exist also between the temperature measured in the ear with an ear thermometer and the average, maximum and minimum temperature of the forehead. This seems surprising as the temperature distribution on the forehead shows great heterogeneity. Therefore, the measurement of the forehead temperature does not seem to be a good parameter for determining the core body temperature. The temperature of the forehead of a person entering a building can be significantly influenced by, e.g., headgear, hair covering the forehead, sweat, and the weather. However, the obtained results clearly showed the highest correlation for the mean forehead temperature with the established gold standard (T_ET_ vs. T_Fa_ r = 0.62, *p* < 0.0001).

Previous thermovision studies have shown that the measurement taken in the inner corners of the eyes is the closest and, at the same time, the most reliable to deep body temperature [11,17,18]. This measurement is similar to that in the armpit with a contact thermometer. Therefore, based on this knowledge, international guidelines for the measurement of the core temperature of the human body using thermovision of the inner corners of the eyes have been developed [19]. Although it is known that the temperature of the inner corner of the eye is consistently the warmest area on the head and the most suitable place for use in detecting fever [17,18]. However, the temperature of an inner corner or other place on the skin is not the same as the core body temperature and will always be slightly lower. At the same time, its value is unfortunately often variable. Recently, it has also been shown that climatic conditions (temperature or environmental humidity) also influence the temperature value in the inner part of the eye [17]. For example, it was shown that an increase in air temperature from 15.5 °C to 26.6 °C caused an increase in the temperature in the inner corners of the eye from 35.7 °C to 37.6 °C, although the core body temperature remained unchanged [17,19].

To sum up, the obtained results allowed for the determination of a thermovision measurement protocol including correlation coefficients between temperature and climatic parameters. This was the first step in the remote core body temperature measurement that will lead to the formation of correlation equations. Thanks to these equations, it will be possible to obtain a high agreement of the core temperature value with that measured by contact thermometry. High concordance of the calculated body temperature with the core temperature, thus adopting an isotherm threshold above 37 °C, would be the most appropriate screening measurement. Moreover, it seems that the remote fever assessment procedure should take into account at least two equations for the body’s core temperature. This will allow the use of thermography as a screening method, allowing the search for people with increased body temperature. 

In the face of a pandemic, research using thermovision seems to be particularly useful [9,20]. However, it should be emphasized that thermovision as a screening method may have some limitations. There may be situations where infectious people may not have elevated core body temperature and therefore will not be detected by the screening system. This may be the case with a newly infected person. The virus may still be in the incubation phase because the immune response has not yet been activated. A misjudgment in the proposed screening system may occur while the person is still sick but no longer has symptoms of fever. Likewise, a person who has taken pharmaceutical measures to reduce fever will not be detected. Nevertheless, it is believed that fever screening can reduce disease spreading statistics by up to 50% [17].

## 5. Conclusions

The use of thermal imaging to detect persons with elevated body temperature has shown very promising results. Remote thermographic measurement of body temperature and taking into account the appropriate values of coefficients and algorithms can be a useful criterion for assessing core body temperature. In the near future, the evaluation of the core body temperature based on developed correlation equations could be very quickly and accurately measured with a thermal imaging camera. Thus, thermovision can be used as a screening procedure of potentially virus-infected people who want to enter public institutions.

## Figures and Tables

**Table 1:** Averaged climatic data and temperature values obtained from the analysis of thermograms and measured with thermometers.

Parameters [Unit]	Mean	Standard Deviation	Median	Minimum Value	Maximum Value
**T_O_ [°C]**	13.40	3.17	13.00	10.10	17.00
**T_I_ [°C]**	22.19	1.35	22.30	20.10	23.90
**H_O_ [%]**	77.50	6.09	79.00	68.00	88.00
**V_W_ [m/s]**	1.26	0.96	0.83	0.40	2.78
**T_F max_ [°C]**	32.92	1.26	33.10	29.30	35.40
**T_F min_ [°C]**	30.36	1.70	30.75	25.70	33.40
**T_Fa_ [°C]**	31.57	1.52	31.90	27.30	34.60
**T_CE max_ [°C]**	35.05	0.75	35.10	33.40	36.60
**T_M max_ [°C]**	36.09	1.05	36.25	32.00	38.20
**T_E max_ [°C]**	35.19	1.06	35.35	31.80	37.30
**T_AR_ [°C]**	35.82	0.53	35.80	34.40	36.90
**T_ET_ [°C]**	35.67	0.75	35.80	34.00	37.20

T_O_—the temperature outside the building; T_I_—temperature inside the building; H_O_—humidity outside; V_W_—wind speed; T_F max_—maximum temperature of the forehead; T_F min_—minimum temperature of the forehead; T_Fa_—average forehead temperature; T_CE max_—maximum temperature of the corners of the eyes; T_AR_—the temperature in the armpit; T_E max_—maximum temperature in the ear; T_M max_—temperature in the mouth; T_ET_—temperature in the ear measured with ear thermometer.

**Table 2:** Correlations between external factors and temperature values. The statistically significant differences were marked with red color.

		Correlations	
External Factors [Unit]	Temperature Measurements [°C]	r	*p*
**T_O_ [°C] vs.**	T_Fmax_	0.17	0.054
	T_Fmin_	0.25	0.004
	T_Fa_	0.24	0.006
	T_CEmax_	0.18	0.039
	T_Mmax_	0.15	0.098
	T_Emax_	0.22	0.013
	T_AR_	0.00	0.999
	T_ET_	0.12	0.160
**H_O_ [%] vs.**	T_Fmax_	0.20	0.021
	T_Fmin_	0.25	0.004
	T_Fa_	0.26	0.003
	T_CEmax_	0.20	0.025
	T_Mmax_	0.16	0.061
	T_Emax_	0.21	0.017
	T_AR_	−0.11	0.215
	T_ET_	0.14	0.101
**V_W_ [m/s] vs.**	T_Fmax_	0.15	0.079
	T_Fmin_	0.25	0.005
	T_Fa_	0.23	0.010
	T_CEmax_	0.22	0.013
	T_Mmax_	0.21	0.016
	T_Emax_	0.16	0.077
	T_AR_	−0.12	0.192
	T_ET_	0.10	0.257

**Table 3:** Correlations between temperature values in the ear measured with the ear thermometer and temperature values of selected parts of the face measured with the thermal imaging camera. The statistically significant differences were marked with a red color. The highest Spearman correlation coefficient values are marked with stars.

		Correlations	
Temperature Measurements [°C]		r	*p*
**T_ET_ vs.**	T_Fmax_	0.57	<0.0001
	T_Fmin_	0.61	<0.0001
	T_Fa_	0.62 *	<0.0001
	T_CEmax_	0.54	<0.0001
	T_Mmax_	0.27	0.016
	T_Emax_	0.65 *	<0.0001
	T_AR_	0.15	0.090

## Data Availability

Data sharing is not applicable to this article.

## References

[1] Chang M.C., Park Y.K., Kim B.O., Park D. (2020). Risk factors for disease progression in COVID-19 patients. BMC Infect Dis..

[2] World Health Organization Coronavirus Disease 2019 (COVID-19) Situation Reports. https://www.who.int/publications/m/item/weekly-epidemiological-update-on-covid-19---15-march-2022.

[3] Wang Y., Wang Y., Chen Y., Qin Q. (2020). Unique epidemiological and clinical features of the emerging 2019 novel coronavirus pneumonia (COVID-19) implicate special control measures. J. Med. Virol..

[4] Porcheddu R., Serra C., Kelvin D., Kelvin N., Rubino S. (2020). Similarity in Case Fatality Rates (CFR) of COVID-19/SARS-COV-2 in Italy and China. J. Infect. Dev. Ctries..

[5] Shi S., Qin M., Shen B., Cai Y., Liu T., Yang F., Gong W., Liu X., Liang J., Zhao Q. (2020). Association of cardiac injury with mortality in hospitalized patients with COVID-19 in Wuhan, China. JAMA Cardiol..

[6] Zhou F., Yu T., Du R., Fan G., Liu Y., Liu Z., Xiang J., Wang Y., Song B., Gu X. (2020). Clinical course and risk factors for mortality of adult inpatients with COVID-19 in Wuhan, China: A retrospective cohort study. Lancet.

[7] Barry A., Apisarnthanarax S., O’Kane G.M., Sapisochin G., Beecroft R., Salem R., Yoon S.M., Lim Y.-S., Bridgewater J., Davidson B. (2020). Management of primary hepatic malignancies during the COVID-19 pandemic: Recommendations for risk mitigation from a multidisciplinary perspective. Lancet Gastroenterol. Hepatol..

[8] Allali S., Beddok A., Kirova Y. (2022). Is cancer a prognostic factor for severe COVID-19, especially for breast cancer patients?. Cancer Radiother..

[9] Abuzairi T., Imaniati Sumantri N., Irfan A., Maulana Mohamad R. (2021). Infrared thermometer on the wall (iThermowall): An open source and 3-D print infrared thermometer for fever screening. HardwareX.

[10] Podbielska H., Skrzek A. (2014). Biomedyczne Zastosowania Termowizji.

[11] Avner J.R. (2009). Ostra Gorączka. Pediatr. Dyplomie.

[12] Sathyamoorthy D., Yunus A.R.M. (2011). Thermographic mass blind fever screening: A review of the effectiveness of correlation tests and operations. Def. ST Tech. Bull..

[13] Chiang M.F., Lin P.W., Lin L.F., Chiou H.Y., Chien C.-W., Chu S.-F., Chiu W.-T. (2008). Mass screening of suspected febrile patients with remote-sensing infrared thermography: Alarm temperature and optimal distance. J. Formos. Med. Assoc..

[14] Ng E.Y., Acharya R.U. (2009). Remote-sensing infrared thermography. IEEE Eng. Med. Biol. Mag..

[15] Bitar D., Goubar A., Desenclos J.C. (2009). International travels and fever screening during epidemics: A literature review on the effectiveness and potential use of non-contact infrared thermometers. Eurosurveillance.

[16] Ng E.Y.K., Annonos P., Rossi M., Pham T.D., Falugi C., Bussing A., Koukou M. (2010). Advanced Integrative Thermography in Identification of Human Elevated Temperature. Advances in Biomedical Research.

[17] Mercer J.B., Ring E.F.J. (2009). Fever screening and infrared thermal imaging: Concerns and guidelines. Thermol. Int..

[18] Ring F. (2007). Pandemic: Thermography for fever screening of airport passengers. Thermol. Int..

[19] Vardasca R., Marques A.R., Diz J., Seixas A., Mendes J., Ring E.F.J. (2017). The influence of angles and distance on assessing inner-canthi of the eye skin temperature. Thermol. Int..

[20] Nguyen A.V., Cohen N.J., Lipman H., Brown C.M., Molinari N.-A., Jackson W.L., Kirking H., Szymanowski P., Wilson T.W., Salhi B. (2010). Comparison of 3 infrared thermal detection systems and self-report for mass fever screening. Emerg. Infect. Dis..

